# Transcriptomic and Proteomic Analyses Reveal the Diversity of Venom Components from the Vaejovid Scorpion *Serradigitus gertschi*

**DOI:** 10.3390/toxins10090359

**Published:** 2018-09-05

**Authors:** Maria Teresa Romero-Gutiérrez, Carlos Eduardo Santibáñez-López, Juana María Jiménez-Vargas, Cesar Vicente Ferreira Batista, Ernesto Ortiz, Lourival Domingos Possani

**Affiliations:** 1 Departamento de Medicina Molecular y Bioprocesos, Instituto de Biotecnología, Universidad Nacional Autónoma de México, Avenida Universidad 2001, Apartado Postal 510-3, Cuernavaca, Morelos 62210, Mexico; teresaro@ibt.unam.mx (M.T.R.-G.); santibanezlo@wisc.edu (C.E.S.-L.); jimenez@ibt.unam.mx (J.M.J.-V.); 2 Department of Integrative Biology, University of Wisconsin–Madison, Madison, WI 53706, USA; 3 Laboratorio Universitario de Proteómica, Instituto de Biotecnología, Universidad Nacional Autónoma de México, Avenida Universidad 2001, Apartado Postal 510-3, Cuernavaca, Morelos 62210, Mexico; fbatista@ibt.unam.mx

**Keywords:** proteome, *Serradigitus gertschi*, scorpion, transcriptome, venom

## Abstract

To understand the diversity of scorpion venom, RNA from venomous glands from a sawfinger scorpion, *Serradigitus gertschi*, of the family Vaejovidae, was extracted and used for transcriptomic analysis. A total of 84,835 transcripts were assembled after Illumina sequencing. From those, 119 transcripts were annotated and found to putatively code for peptides or proteins that share sequence similarities with the previously reported venom components of other species. In accordance with sequence similarity, the transcripts were classified as potentially coding for 37 ion channel toxins; 17 host defense peptides; 28 enzymes, including phospholipases, hyaluronidases, metalloproteases, and serine proteases; nine protease inhibitor-like peptides; 10 peptides of the cysteine-rich secretory proteins, antigen 5, and pathogenesis-related 1 protein superfamily; seven La1-like peptides; and 11 sequences classified as “other venom components”. A mass fingerprint performed by mass spectrometry identified 204 components with molecular masses varying from 444.26 Da to 12,432.80 Da, plus several higher molecular weight proteins whose precise masses were not determined. The LC-MS/MS analysis of a tryptic digestion of the soluble venom resulted in the *de novo* determination of 16,840 peptide sequences, 24 of which matched sequences predicted from the translated transcriptome. The database presented here increases our general knowledge of the biodiversity of venom components from neglected non-buthid scorpions.

## 1. Introduction

The family Vaejovidae, which currently includes nearly 240 species [1], is subdivided into three subfamilies: Smeringurinae, Syntropinae, and Vaejovinae [1,2]; however, four genera (i.e., *Gertschius*, *Serradigitus*, *Stahnkeus*, and *Wernerius*) are *incertae sedis*, which means that their morphological characteristics do not allow their inclusion into any subfamily as currently stated. The genus *Serradigitus* (Stahnke, 1974) is represented by 14 species in Mexico and five more in the United States of America (USA) [2]. Most of these species are distributed within the Baja California Peninsula [2], and are considered as lithophilous species, because they live in rocky environments, such as cliff, wall, or stone crevices [3]. Encounters with humans are rare due to their habitat preferences, and no reports have been filed on human intoxication from these species, which could possibly mean that their venom is not toxic to humans or that they have not been correctly identified in envenomation cases. The venom composition of species belonging to genus *Serradigitus* (sawfinger scorpions) is still unknown. To the best of our knowledge, there are no reports on the possible venom components from this genus.

In recent years, scorpion venom gland transcriptome analyses of vaejovid scorpion species have shed some light on the biodiversity of components of the venom of these scorpions. The first vaejovid scorpion venom components studied were isolated from *Vaejovis mexicanus*, which is a member of subfamily Vaejovinae. They were Vejovine, a non-disulfide bridged peptide (NDBP) [4], and two potassium channel toxins (Vm23 and Vm24) [5,6]. Later, cDNA libraries were constructed for four vaejovid species: *Mesomexovis subcristatus* (*Vaejovis subcristatus*), *Mesomexovis variegatus* (*Vaejovis punctatus*), *Thorellius atrox* (*Vaejovis intrepridus*), and *Vaejovis mexicanus* [7]. These studies showed that the biodiversity of NDBPs was high in these species’ venoms. More recently, the first high-throughput transcriptomic and proteomic analyses for the Vaejovidae species were performed for *T. atrox* [8] and *Paravaejovis schwenkmeyeri* [9], suggesting that the diversity of the ion channel toxin components found in previous studies is similar. Unfortunately, the Vaejovidae family is among the most diverse scorpion families, and the diversity of venom components is known only from a handful of species restricted to two (of three) subfamilies and four (out of 25) genera included in this family, preventing further studies on venom evolution. They revealed the large diversity of venom components in these species, including transcripts potentially coding for a novel class of biomolecules according to their conserved domain (omegascorpins, insulin-like growth factor binding protein (IGFBP), La1-like-peptides). Both *T. atrox* and *P. schwenkmeyeri* belong to the subfamily Syntropinae. In contrast to other scorpion families, a rigorous analysis of the phylogeny of the subfamily Syntropinae has been performed only recently [10]. However, this is not the case for the other two subfamilies within the family Vaejovidae, for which deeper phylogenetic studies are required. Moreover, several genera (e.g., genus *Serradigitus*) remain *incertae sedis*, as indicated above. This issue, together with the inherent interest that the study of the venom composition for these neglected orphan taxa represents, prompt for the high-throughput sequence exploration of their venoms.

In the present work, we used transcriptomic and proteomic analyses to identify the components of venom of the scorpion *Serradigitus gertschi*, which is a relatively small scorpion (20–35 mm) that can be found in California in the USA and in the Baja California Peninsula in Mexico [11]. This species lives under logs or small objects on the ground in arid areas. They prey on small arthropods by leaving their diurnal shelter in early evenings, exposing their sensory receptors until prey stimulates them [12]. We report 119 annotated transcripts that putatively code for venom components on similarity, including ion channel-acting toxins and other venom-specific peptides and proteins. The proteomic analysis revealed that 24 of the encoded peptides are indeed present in the venom, thus validating the results of the transcriptomic analysis. This contribution is the first report toward the identification of the venom components of this enigmatic genus whose phylogenetic status remains unclear. The generated sequence database will aid in the definitive taxonomic classification and future research aimed at identifying venom evolution within the Vaejovidae family.

## 2. Results and Discussion

### 2.1. Serradigitus Gertschi Venom Gland Global Transcriptome Analysis

After sequencing, assembly and rRNA and adapter cleaning, 17,363,454 reads were obtained. The sequences were uploaded to the European Nucleotide Archive (ENA) under project PRJEB27910. The reads were assembled into 84,835 transcripts with an N50 of 646 bp. From them, a total of 21,410 transcripts were identified as potentially coding for peptides/proteins with sequence similarity to previously reported sequences.

**Figure 1:**
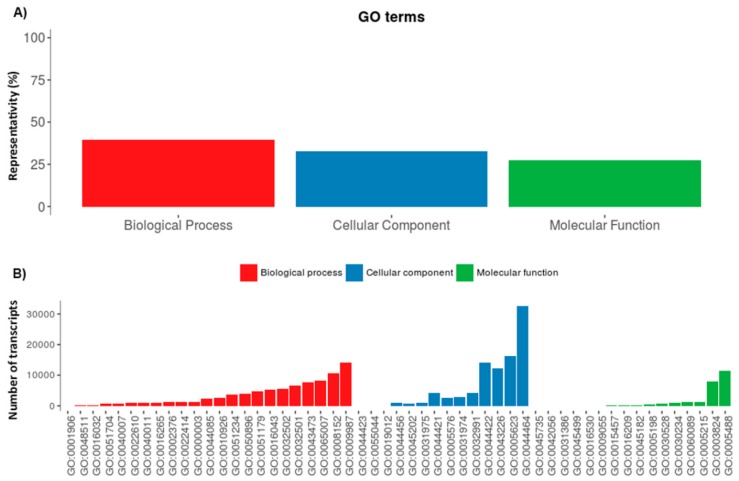
Gene Ontology distribution of the annotated transcripts. (**A**) Distribution of the annotated sequences from the venom gland transcriptome of *S. gertschi* according to Gene Ontology (GO) terms. (**B**) Distribution of the most represented categories within each GO term (GO terms are shown). In GO, each term has defined relationships to one or more other terms in the same domain, and sometimes to other domains; therefore, the apparent number of transcripts in B is larger than the number of annotated transcripts.

Initial work for the classification of the annotated transcripts was done by use of the Gene Ontology (GO) terms. At the broadest level of ontology, 39.7% of the transcripts were identified as belonging to the biological process, 32.8% were identified as belonging to the cellular component, and 27.5% were identified as belonging to the molecular function categories. This distribution and the details of the most abundant GO terms within each category are shown in Figure 1.

**Figure 2:**
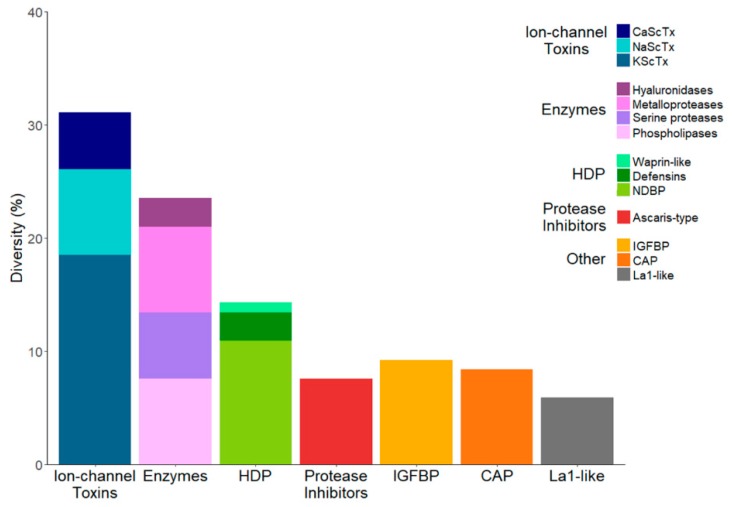
Distribution of the annotated *S. gertschi* transcripts according to the putative function/activity of the encoded peptides/proteins. The diversity is the number of different unique transcripts within each category and subcategory, and does not consider their relative abundance in the transcriptome. HDP: Host defense peptides; NDBP: Non-disulfide rich peptides; IGFBP: Insulin-like growth factor binding protein; CAP: Cysteine-rich secretory proteins, antigen 5, and pathogenesis-related 1 protein.

A subgroup of 119 annotated transcripts, for which the translated amino acid sequences show sequence similarities to venom components, were identified. Among these components are toxins proposed to act on Na^+^, K^+^ and Ca^2+^ ion channels, La1-like peptides, and host defense peptides (HDP) of the defensin family. Non-disulfide bridged peptides (NDBP) were also present, comprising members of families 2, 3, and 4. Larger proteins were also represented, including enzymes (phospholipases, metalloproteases, hyaluronidases, and serine proteases), protease inhibitors, and members of the cysteine-rich secretory proteins, antigen 5, and pathogenesis-related 1 protein (CAP) superfamily. Lastly, some transcripts were identified as potentially coding for other venom components of so far undescribed function. This information is detailed in Supplementary Table S1. The distribution of the annotated transcripts in accordance to their percentage of diversity (i.e., representing the number of unique transcripts that belong to each category or subcategory, but not their relative abundance in terms of read frequencies) is represented in Figure 2.

### 2.2. The Repertoire of Venom-Specific Transcripts in S. gertschi

#### 2.2.1. Ion Channel Toxins

The ion channel-acting toxins represent the landmark components of scorpion venoms. Toxins acting on Na^+^, K^+^, Ca^2+^, and Cl^−^ ion channels have been described as constituents of many scorpion venoms. They are the components that have historically drawn the main research efforts, if only for their relevant role in human intoxication. Their interaction with ion channels results in a cascade of physiological events that, in the most severe cases, can lead to death. The Na^+^ and K^+^ channel toxins are the main culprits of the intoxication symptoms. Therefore, it is expected that in the venoms of the most dangerous scorpion species, e.g., those belonging to the family Buthidae, they would be the most abundant and diverse components, while being less represented in non-Buthidae scorpion venoms. This has been documented in previous proteomic and transcriptomic analyses. The toxic fraction in most buthids is high (ranging from 66% to 85% of the venomous peptides reported [13,14,15,16]), while in non-buthid scorpions, it is much lower (from 9% to 20% [8,9,14,17,18,19,20]).

Given the above, and that *S. gertschi* is a non-buthid species, it came as a surprise that the subgroup of transcripts potentially coding for ion channel toxins was the most diverse in the transcriptome by a significant extent (31.2% of all transcripts, see Figure 2). In previous high-throughput analyses of non-buthid species, this subgroup was either outnumbered by other venom components (as in *T. atrox* and *M. gertschi*, for which enzymes were the most diverse [8,18]) or on par with those other components (as in *Superstitionia donensis* and *P. schwenkmeyeri* [9,19]. The reason for this observation remains to be determined. One trait that is specific for this genus is that it is morphophysiologically adapted to a lithophilous microhabitat (see Figure 9 in Cid-Uribe et al. [9] for comparison), which could have driven the evolution of the venom components in *S. gertschi* in this particular direction. Though not to the extent observed in buthid species, the proportional diversity of the components putatively coding for ion channel toxins in *S. gertschi* is the highest of all of the reported non-buthid species, to the best of our knowledge.

##### Sodium Channel Toxins

Toxins affecting the gating mechanism of Na^+^-channels (NaScTx) are responsible for the neurotoxic symptoms during envenomation [21]. Na^+^-channel toxins were divided in two subtypes. Alpha toxins (α-NaScTx) bind to receptor site 3 of voltage-dependent Na^+^-channels, inhibiting the activation process of the channel. Beta toxins (β-NaScTx) bind to receptor site 4 and shift the activation potential of the ion channel to more negative values, as seen in the review by Gurevitz [22].

**Figure 3:**
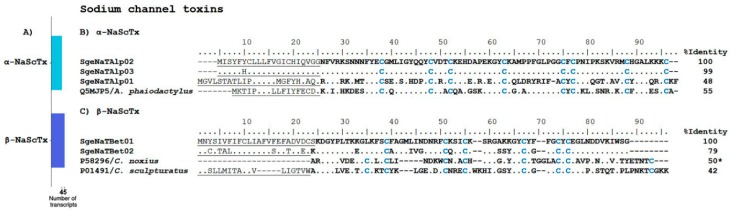
Putative Na^+^ channel-specific toxins encoded by *S. gertschi* transcripts. (**A**) Number of different transcripts putatively coding for α-NaScTx and β-NaScTx. The number of transcripts is the number of unique sequences that putatively encodes different proteins of this subcategory. (**B**) The translated transcripts putatively coding for ion channel toxins similar to phaiodotoxin. (**C**) Transcript-derived partial peptide sequences coding for β-NaScTx aligned to their closest matches by BLAST. Dots indicate sequence identity; dashes indicate gaps. The predicted signal peptides are underlined, the mature peptides are in bold typeface, and the cysteines are highlighted in blue. Reference proteins include their UniProt identifiers and the species’ name. The identity percentages (%I) in the alignments were calculated based on the precursor sequences, except where indicated by an asterisk (*), for which only the mature sequence was considered.

In this transcriptome analysis, we found nine transcripts putatively coding for Na^+^-channel toxins, of which, four transcripts correspond to α-NaScTx and five correspond to β-NaScTx (Figure 3A). Of the former, three precursors showed sequence similarity to the precursor of phaiodotoxin (UniProt Q5MJP5), an α-NaScTx from the scorpion *Anuroctonus phaiodactylus* (now known as *Anuroctonus pococki bajae* [23], see Figure 3B. An amount of 0.5 µg of phaiodotoxin per animal causes paralysis in crickets, and 1 μg causes death [24]. Otherwise, phaiodotoxin is not toxic to mammals, even at doses of 100 µg per mouse [24]. The fourth putative α-NaScTx that was found had sequence similarity to venom peptide HtUy2 (UniProt A0A1B3IJ50), which was derived from a transcript from *Hadogenes troglodytes*, and whose venom is only mildly toxic to mice [17]; see Supplementary Table S1. Five partial transcript sequences potentially coding for β-NaScTx were found in the transcriptome of *S. gertschi* (see Supplementary Table S1). Figure 3C shows the alignment of the two sequences with the best E-values (cut-off point was 1 × 10^−4^), as determined by Blastp, with their best matches. Their closest matches were Cn11 (UniProt P58296), an ion channel toxin found in the venom of the scorpion *Centruroides noxius*, and the precursor of CsEI from *Centruroides sculpturatus* (UniProt P01491). Cn11 is toxic to crustaceans, mildly toxic to crickets, and non-toxic to mammals [25]. CsEI is lethal to chickens and mildly toxic to crickets, but it is not toxic to mice [26]. As indicated, the best matches for the putative NaScTx from *S. gertschi* with the best E-values correspond to ion channel toxins that have been experimentally proven to be non-toxic to mammals (even though the reference β-NaScTx belongs to buthid scorpions). This is an indirect confirmation of the expected non-toxicity of *S. gertschi* to mammals, including humans.

##### Potassium Channel Toxins

The scorpion toxins that affect K^+^ ion channels (KScTx) are, essentially, blockers of these channels. They are peptides that are 20–75 amino acids long [27], and have been divided into six subfamilies (α-, β-, γ-, κ-, λ- and ε-KScTx) based on their primary sequence and disulfide bond connectivity, as reviewed in Jiménez-Vargas et al. [28].

**Figure 4:**
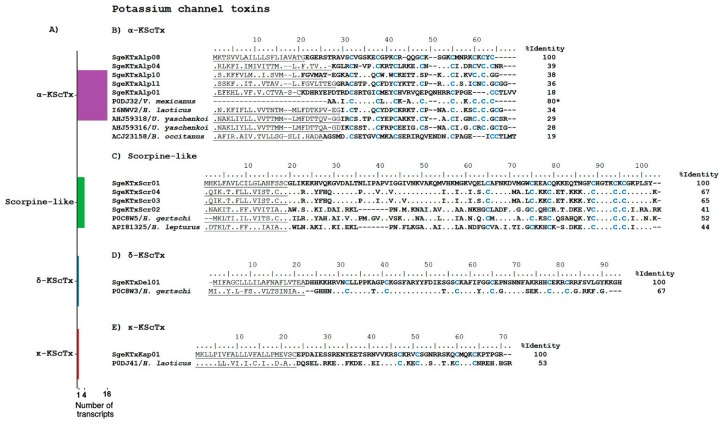
Overview of transcripts related to K^+^ channel toxins in the transcriptome of *S. gertschi*. (**A**) The diversity of sequences putatively coding for peptides belonging to the different K^+^ channel toxin subfamilies. The number of transcripts is the number of unique sequences that putatively encodes to different proteins of this subcategory. (**B**–**E**) Transcript-derived translated precursor sequences for representative α-KScTx, scorpion-like peptides, δ-KScTx and κ-KScTx, aligned to their best matches. Dots indicate sequence identity; dashes indicate gaps. The predicted signal peptides are underlined, the mature peptides are in bold typeface, and the cysteines are highlighted in blue. Reference proteins include their UniProt identifiers and the species’ name. The identity percentages (%I) in the alignments were calculated based on the precursor sequences, except where indicated by an asterisk (*), for which only the mature sequence was considered.

As mentioned above, the analyzed *S. gertschi* transcriptome resulted in highly diverse transcripts coding for putative ion channel toxins. The main contributors to that diversity are transcripts related to KScTx, in particular those of the α-KScTx subfamily. We found 22 transcripts potentially coding for the following KScTx: 16 from the α-KScTx subfamily, four potentially coding for scorpin-like peptides, one for a δ-KScTx, and one for a κ-KScTx (see Supplementary Table S1 and Figure 4). If this transcript diversity reflects the heterogeneity of these ion channel toxins in the expressed venom, then *S. gertschi* should be a rich source of KScTx. The transcripts annotated as KScTx are described below. It has been suggested that peptides displaying sequence similarities to K^+^-channel blocking peptides might be used by scorpions as insecticides [29]. Thus, it would be not surprising to find so many different peptides within this category for a lithophilous scorpion *S. gertschi,* which lives in such harsh conditions, as earlier described.

The α-KScTx subfamily peptides are characterized by the cysteine-stabilized α/β fold (CSαβ), and are high-affinity blockers of the Kv1 family and the BK K^+^ channels [28]. We found 16 transcript sequences related to this family (Supplementary Table S1): SgeKTxlAlp01 and SgeKTxAlp08 showing similarity to the precursors named a ‘potassium channel toxin’ (UniProt API81324 and API81322), respectively, from *Hemiscorpius lepturus* [30]; SgeKTxAlp02 and SgeKTxAlp06, showing similarity to the precursor named a ‘potassium channel toxin’ (UniProt AHJ59316) from *Urodacus. yaschenkoi* [31]; SgeKTxAlp03 and SgeKTxAlp04, showing similarity to the precursor named a ‘potassium channel toxin’ (UniProt AHJ59318) from *U. yaschenkoi* [31]; SgeKTxAlp05 and SgeKTxAlp07, showing similarity to the K^+^-channel toxin Vm23 (UniProt P0DJ32) isolated from the venom of *V. mexicanus*, which selectively and irreversible binds and blocks Kv1.3 channels of human T-lymphocytes and weakly blocks Kv1.2 channels [32]; SgeKTxAlp09, with similarity to the transcript ‘potassium channel toxin’ (UniProt API81322) from *H. lepturus*; SgeKTxAlp10, SgeKTxAlp11, and SgeKtxAlp13, showing similarity to the precursor Tx3 from *Heterometrus laoticus* (UniProt AFB73769); SgeKTxAlp12 and SgeKTxAlp14, with similarity to the precursor ‘potassium channel toxin’ (UniProt A0A0A0PI37); SgeKTxAlp15 shows similarity with the precursor potassium channel toxin alpha-KTx 6.8 from *Opistophthalmus carinatus* (UniProt Q6XLL7); and SgeKTxAlp16, with similarity to HtKTx3 (UniProt AOF40181) from *H. troglodytes*. Figure 4B shows the translated sequences of several of these transcripts aligned to the translated sequences of related precursors and an ion channel toxin.

Initially, the scorpine-like peptides were classified as members of the β-KScTx family; however, they are now considered as an independent subfamily of the KScTx [19]. They are long peptides containing 59–75 amino acids stabilized by disulfide linkages [33] with two domains: one showing cytolytic activity, and another with K^+^ channel-blocking properties [34]. Figure 4C shows the precursors of the possible scorpine-like peptides from *S. gertschi* aligned to translated precursors from other scorpion species with sequence similarity, namely: Hg-scorpine-like 2 (UniProt P0C8W5), from *Hadrurus gertschi* [35], and A0A1L4BJ43_HEMLE (UniProt A0A1L4BJ43), from *H. lepturus* [30]. SgeKTxScr03 and SgeKTxScr04 encode very similar amino acid sequences, with changes in only three amino acids (positions 40, 46, and 88 in the alignment in Figure 4C).

δ-KScTx is a subfamily of the KScTx with a Kunitz-type fold [36]. Functionally, these ion channel toxins inhibit protease activity and block voltage-dependent potassium channels [36]. δ-KScTx has been found in other venomous animals, such as cone snails, anemones, spiders, and snakes [37]. We found a single transcript coding for a putative δ-KScTx (Supplementary Table S1). The alignment of its putative precursor with the precursor of Hg1 (UniProt P0C8W3) from *H. gertschi* is shown in Figure 4D. Hg1 inhibits the activity of trypsin, blocks the murine Kv1.3 channel, and has weak activity against the murine Kv1.1, the human Kv1.2, and the human KCa2.3 [38].

The subfamily of potassium toxins κ-KScTx is structurally characterized by two α-helices connected with two disulfide bonds: the so-called cysteine-stabilized α/α motif (CSαα) [39]. We found one sequence coding for a possible precursor with 53% similarity to the precursor of HelaTx1 (UniProt P0DJ41), which is an ion channel toxin from *H. laoticus* (Figure 4E) and a blocker of the Kv1.1 and Kv1.6 channels [40].

##### Calcium Channel Toxins

**Figure 5:**
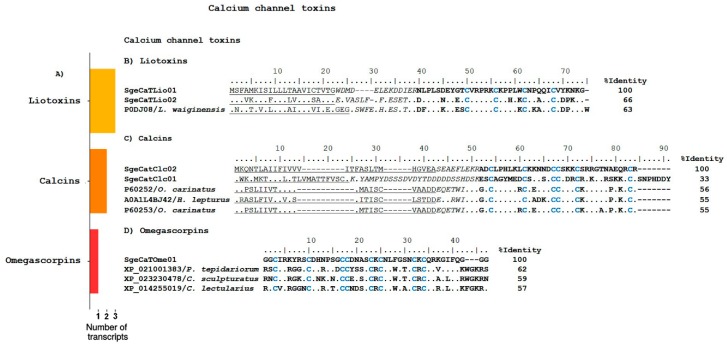
Putative Ca^2+^ channel toxins identified by the transcriptome analysis of *S. gertschi*. (**A**) Distribution of the different transcripts putatively coding for calcins, liotoxins, and omegascorpins. The number of transcripts is the number of unique sequences that putatively encodes to the different proteins of this subcategory. (**B**) The CDS from the transcripts coding for putative liotoxins aligned to reference precursor sequences. (**C**) Transcript-derived precursor sequences coding for calcins together with previously reported similar precursor sequences. (**D**) Transcript-derived mature predicted sequences that putatively code for omegascorpins, aligned with their best matches according to BLAST. The predicted signal peptides are underlined, the propeptides are in italics, the mature peptides are in bold typeface, and the cysteines are highlighted in blue. Reference proteins include their UniProt identifiers and the species’ name. The identity percentages (%I) in the alignments were calculated based on the precursor sequences for the liotoxins and calcins, or the mature sequences for the omegascorpins.

Ca^2+^ channels modifiers can be divided in two main groups: those that affect ligand-gated channels (including scorpion liotoxins and calcins) [41,42], and those acting on voltage-sensitive channels. In the transcriptome analysis performed, six sequences were found that code for possible Ca^2+^ channel toxins (CaScTx), as judged by sequence similarity with other peptides with this activity (Figure 5A).

Liotoxins constitute a group of CaScTx that are structurally characterized by a disulfide-directed hairpin (DDH) fold, stabilized by two disulfide bridges, which is considered the evolutionary precursor of the inhibitor cystine knot (ICK) motif [43]. These proteins affect the activity of ryanodine-sensitive Ca^2+^-release channels RyR1 and RyR2 with high affinity [29]. We found two sequences coding for putative liotoxins, SgeCaTLio01 and SgeCaTLio02, which are similar to those found in other scorpions. The translated precursors are shown in Figure 5B, and are aligned with the precursor of Phi-LITX-Lw1a (UniProt P0DJ08) from *Liocheles waigiensis*. It is interesting to notice that although differences in sequence can be found throughout the whole precursor, the signal peptides and the mature regions appear to be better conserved. Along with the cysteines, the mature peptides preserve the basic residues at positions Arg52, Arg54, Lys55, Lys56, and Lys73, as shown in the alignment. Arg54 has been shown to be important for Phi-LITx-Lw1a lethality in crickets [29].

Calcins are CaScTx that bind with high affinity to RyRs and increase their activity by inducing the appearance of a subconductance state [42]. They are structurally characterized by an ICK motif, and stabilized by three disulfide bridges [42]. We found three transcripts that code for possible calcins (see Supplementary Table S1 and Figure 5C). SgeCatClc01 encodes a precursor that shows sequence similarity to the precursors of Opicalcin-1 (UniProt P60252) and Opicalcin-2 (UniProt P60253) from *Opistophthalmus carinatus* [44]. SgeCatClc02 and SgeCatClc03 encode putative calcins with sequence similarity to Hemicalcin (UniProt API81327) from *H. lepturus*, which is a calcin known to act on the RyR1 receptor, increasing ryanodine binding and triggering Ca^2+^ release from the sarcoplasmic vesicles [45]. It is important to note that the predicted mature peptide derived from SgeCatClc01 has the ICK structural motif and the distinctive functional domain of the calcins, although both the propeptide and the mature regions are longer than those previously described for other calcins (Figure 5C). SgeCatClc03 is a partial sequence, and was not included in the alignment of Figure 5C to avoid affecting the identity percentages.

##### Omegascorpins

These putative peptides have never been isolated from scorpion venoms, but transcripts coding for them have recently emerged from several scorpion venom gland transcriptomic analyses [8,9,18,30]. Their closest matches by sequence similarity, while conserving the cysteine array, are the omega-agatoxins, which are peptides found in the venom of the funnel-web spider *Agelenopsis aperta*. For this reason, these putative scorpion peptides have been named ‘omegascorpins’ [8]. Omega-agatoxins are inhibitors of the neuronal, voltage-activated, P/Q-type Ca^2+^ channel [46], so by analogy, we have included the omegascorpins within the CaScTx class, even though their specific activity remains to be experimentally determined.

In the transcriptome analysis of the *S. gertschi* venom gland, we found one sequence annotated as coding for a putative omegascorpin. Figure 5D shows the alignment of the expected mature peptide translated from this transcript, SgeCaTOme01, with the translated mature sequences derived from the genomes of the spider *Parasteatoda tepidariorum* (GenBank XP_021001383), the scorpion *C. sculpturatus* (GenBank XP_023230478), and the spider *Cimex lectularius* (GenBank XP_014255019).

#### 2.2.2. Host Defense Peptides

Defensins are small, cysteine-rich (DBP), host defense peptides that are found in all living species, including mammals, plants, and fungi, among others [47]. They play an important role in the innate immune system, having antimicrobial, chemotactic, and regulatory activities [48]. They display antimicrobial activity against a broad range of microorganisms, fungi, and viruses [49].

**Figure 6:**
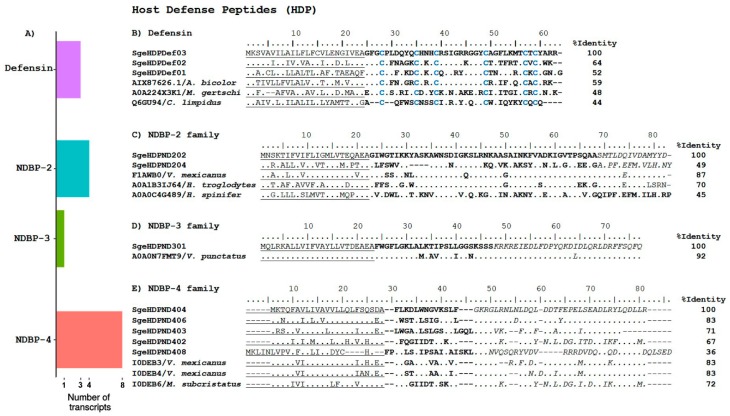
Putative host defense peptides (HDPs) encoded by *S. gertschi* transcripts. (**A**) Diversity of HDP-coding transcripts. The number of transcripts is the number of unique sequences that putatively encodes to different proteins of this subcategory. (**B**) The translated putative defensins aligned to reference precursor sequences. (**C**–**E**) The translated precursor sequences coding for possible members of the NDBP-2, -3, and -4 subfamilies, respectively, together with previously reported similar precursor sequences. The identity percentages (%I) in the alignments were calculated based on the precursor sequences. The predicted signal peptides are shown underlined, the mature peptides are shown in bold typeface, and the propeptides are shown in italics.

We found three sequences that putatively code for defensins (Figure 6A). Their translated ORFs are shown in Figure 6B; they are aligned to the reference precursors of Defensin-1 (UniProt AIX87626), which is identified in the cDNA library of the scorpion *A. bicolor* [13], Defensin-1 (UniProt Q6GU94), which is a peptide found in the hemolymph of *Centruroides limpidus* [50], and the putative Defensin-1 (UniProt A0A224X3K1), which is found in the transcriptome of *M. gertschi* [18].

Waprins are also considered DBP members of the HDP category. They are relatively large (c.a. 50 amino acids) peptides with four conserved disulfide bonds. Sequences coding for waprins were first identified in snakes [51], then in the frog *Ceratophrys calcarata* [52], and recently in scorpions [8,9]. Nawaprin was isolated from the venom of the cobra *Naja nigricollis* [53], confirming that waprins are indeed expressed. Recombinant waprins have demonstrated antimicrobial activities against gram-negative and gram-positive bacteria, as well as fungi [52]. They can also function as protease inhibitors [54]. In this transcriptome analysis, we found one partial transcript that putatively codes for a waprin. The partial CDS from SgeHDPWap01 shares sequence similarity with waprin–Enh1-like (GenBank XM_023384632.1), which is a sequence found in the genome analysis of *C. sculpturatus*.

##### Non-Disulfide Bridged Peptides

The venoms of non-buthid scorpions are usually rich in NDBP. These peptides lack cysteines, so they adopt a random coil structure in aqueous solutions. When surrounded by less polar membranous or membrane-mimicking environments, they change their conformation to form amphipathic **α**-helixes [55]. They display several biological activities, such as antimicrobial, cytolytic, and anti-inflammatory activities, among others [55], conferring to them a major attraction as potential drug candidates [56].

In this analysis, we found 13 transcripts that code for the possible NDBP of the following groups: four transcripts for the NDBP-2 group, one transcript for the NDBP-3 group, and eight transcripts for the NDBP-4 group (Supplementary Table S1 and Figure 6A).

Members of the NDBP-2 group are considered long-chain multifunctional peptides. They have been reported to display antimicrobial, bradykinin-potentiating, and insecticidal activities [55]. Two of the four transcripts that putatively code for NDBP-2 comprise the complete CDS. They are shown in Figure 6C aligned to the following three reference sequences: the precursor for vejovine (UniProt F1AWB0) from *V. mexicanus*, which is a peptide with antibacterial activity against gram-negative multidrug-resistant strains of *Escherichia coli*, *Pseudomonas aeruginosa*, and *Acinetobacter baumanii*, with MIC values as low as 5 µM [4]; the precursor for HtAPx (UniProt A0A1B3IJ64), which is found in the transcriptome of the scorpion *H. troglodytes* [17]; and Heterin 1 (UniProt A0A0C4G489), which is a transcript found in the transcriptome of the scorpion *Heterometrus spinifer* [20].

The NDBP-3 group includes medium-length (24 to 29 amino acids) cationic antimicrobial peptides [55]. The precursor, derived from the transcript corresponding to this kind of peptides found in this analysis, is shown in Figure 6D, aligned to its best sequence match, the precursor of Amp2 (UniProt A0A0N7FMT9), from the cDNA library of *M. variegatus* [57]. The signal peptide and the propeptide regions are highly conserved, while the sequence divergence is evident in the mature region.

The NDBP-4 are considered short (13 to 19 amino acids) cationic antimicrobial peptides. The precursors for these peptides include a signal peptide, a Lys/Arg-rich mature region, and a propeptide that starts with an amidation signal (GKR). Therefore, they are C-terminal amidated peptides. A broad spectrum of activity was reported for these peptides, both against gram-negative and gram-positive bacteria [57]. The NDBP-4 group was the most diverse within the HDP category in this transcriptome, with eight transcripts matching these characteristics. Figure 6E shows the alignment of the CDS derived from five of the transcripts, with a few reference sequences belonging to the NDBP-4 group (excluded were SgeHDPDN401 and SgeHDPDN407, which lacked part of the CDS, and SgeHDPDN405, which has a mature sequence identical to that of SgeHDPDN404, see Supplementary Table S1). The references are: the precursor of VsCT2 (UniProt I0DEB6) found in the cDNA library of the scorpion *M. subcristatus* [58]; and the precursors of VmCT1 (UniProt I0DEB3) and VmCT2 (UniProt I0DEB4), which are both found in a cDNA library from *V. mexicanus* [58]. Again, the signal peptide and the propeptide regions are the most conserved, while the mature peptides show very low identity.

#### 2.2.3. Enzymes

Enzymes are among the most abundant components in scorpion venoms according to recent findings [18,59]. Here, we report 28 transcripts that putatively code for four types of enzymes: nine metalloproteases, nine phospholipases, seven serine proteases, and three hyaluronidases.

Metalloproteases are commonly found in venomous animals, such snakes [60], spiders [61], Gila monsters [62], and scorpions [63]. Their presence in venoms has been associated with envenomation-related pathologies such as local and systemic hemorrhage, myonecrosis, blistering, hypovolemia, and inflammation [64], mainly in snakes. We found nine transcripts that putatively code for metalloproteinases, of which only one sequence was complete (Supplementary Table S1).

Phospholipases catalyze the hydrolysis of phospholipids [65]. The proposed role for phospholipases in scorpion venoms relates to their ability to damage lipid membranes, allowing the spread of ion channel toxins and other venom components [66]. In the particular case of *H. lepturus*, potent phospholipase D activities have been associated with the highly toxic (even lethal) necrosis activity of the venom [67]. To date, distinct classes of phospholipases have been characterized, including: type A, B1 and B2, C, and D [68]. We identified seven transcripts related to phospholipase A2, one to phospholipase B2, and another for phospholipase D, of which, only SgeEnzPLB01 was complete (Supplementary Table S1).

Hyaluronidases are enzymes that degrade hyaluronic acid. These enzymes are not toxic themselves, but potentiate the effect of ion channel toxins present in venoms, acting as spreading factors [69]. Hyaluronidases have been identified in many animal venoms, such as those of insects [70], arachnids [8,18,19,71,72], and snakes [73]. We found three transcripts that putatively code for hyaluronidases, of which only transcript SgeEnzHya01 was recovered with the complete CDS (Supplementary Table S1).

Serine proteases have been identified mainly in snake venoms, but these enzymes have also been found in the venoms of insects [74], as well as in toad [75] and frog [76] secretions. Cytotoxic and antimicrobial activities were also reported for these enzymes [77,78]. Additionally, it has been reported that snake serine proteases cause hemostatic disequilibrium in prey through their action on several components of the coagulation cascade [75,79]. We found seven partial transcripts that putatively code for serine proteases (Supplementary Table S1).

#### 2.2.4. Protease Inhibitors

These peptides protect the ion channel toxins and other venom components from degradation [36]. These peptides have been identified in several venomous animals such as snakes [80], scorpions [36,81], frogs [82], and insects [83]. Specifically, in scorpion venoms, three types of protease inhibitors have been identified: Kunitz-type [84], Ascaris-type [36], and serpins [8]. We found seven transcripts with complete CDS that putatively code for protease inhibitors, all of which correspond to the Ascaris-type inhibitors (Supplementary Table S1).

#### 2.2.5. Other Venom Components

##### La1-Like Peptides

**Figure 7:**
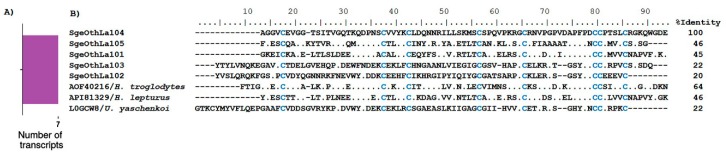
Transcripts related to La1-like peptides encoded by *S. gertschi* transcripts. (**A**) The diversity of transcripts annotated as coding for La1-like peptides. The number of transcripts is the number of unique sequences that putatively encodes to different proteins of this subcategory. (**B**) Transcript-derived mature peptides putatively coding for La1 peptides aligned with their best matches according to BLAST. The mature peptides are in bold typeface and the cysteines are highlighted in blue. Reference proteins include their UniProt identifiers and the species’ name. The identity percentages (%I) in the alignments were calculated based on the precursor sequences.

These peptides are 73–100 amino acids long, are stabilized by four disulfide bridges, and conform a von Willebrand factor type C domain [85]. La1 peptides have been identified in scorpion venoms, as well as in insects and ticks [86]. The first La1 peptide was isolated from the venom of the scorpion *Liocheles australasiae*, which is one of the most abundant components in this venom [86]. Until recently, no biological activity had been ascribed to these peptides, but spermaurin, an La1-like peptide found in the venom of *Scorpio maurus palmatus*, was shown to enhance sperm motility and fertilization rates in mammals [87]. The molecular target for La1-like peptides remains unknown. We found seven transcripts coding for putative La1-like peptides, five of which contained complete CDS (Supplementary Table S1). Thus, La1-like transcripts represent around 8% of the overall diversity of all transcripts coding for venom components in *S. gertschi*. This number is somewhat higher than the reported for other scorpion transcriptomes: 2% in *M. gertschi* and *P. schwenkmeyeri*, 4% in *U. yaschenkoi*, and 5% in *S. donensis* and *T. atrox* [8,19]. Figure 7B shows the alignment of the translated putative mature La1-like peptides from *S. donensis* (only those with complete CDS) in conjunction with HtLa15 (UniProt A0A1B3IJ41), which is a sequence found in the transcriptome analysis of the scorpion *H. troglodytes* [17], ‘Toxin protein’ (UniProt A0A1L4BJ54) from *H. lepturus* [30], and ‘Toxin-like protein 14’ (UniProt L0GCW8), which is a sequence found in the transcriptome analysis of *U. yaschenkoi* [72].

##### CAP Superfamily

Cysteine-rich secretory proteins, antigen 5, and pathogenesis-related 1 proteins (CAP) are a family of secreted proteins that is distributed in animals, plants, and fungi [88]. These proteins have been associated with protease or protease inhibitor activities, ion channel regulation (the cysteine-rich secretory protein domain of Tpx-1 is related to ion channel toxins and regulates ryanodine receptor Ca^2+^ signaling), and extracellular paracrine and endocrine functions [88]. Previously, members of the CAP superfamily were found in reptiles (snakes and lizards) [89] and mammals [90]. Recently, they were also found by transcriptomic and genomic analyses in scorpions (e.g., *Tityus stigmurus* [91], *Tityus bahiensis* [63], *Centruroides hentzi* [15]). We identified 10 transcripts that putatively code for CAP proteins, five of them with complete CDS (Supplementary Table S1).

##### IGFBP Family

The insulin-like growth factor binding protein (IGFBP) family is defined by the structural similarities of their members and their functional ability to bind insulin-like growth factor (IGF) [92]. IGFBPs act as carriers of IGF in biological fluids, and function as modulators of IGF availability and activity. However, their particular function in venoms remains unknown. We found 11 complete CDS that putatively code for IGFBP (Supplementary Table S1), all of which had similarity with different ‘venom toxins’ found in the transcriptome of *H. lepturus* (UniProt API81342, API81343, API81344, API81346, and API81349).

### 2.3. Proteomic Analyses

#### 2.3.1. Mass Fingerprint Results and MS Data Analysis

The soluble fraction of venom from *S. gertschi* was separated using liquid chromatography, and component molecular weights were determined by mass spectrometry. We identified 204 different compounds; these data are shown in Table 1.

**Figure 8:**
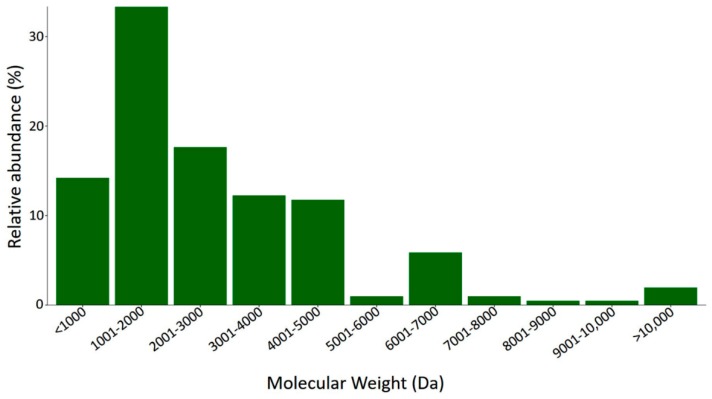
Molecular mass distribution of the components found in the mass fingerprint of the venom of *S. gertschi*.

The distribution of molecular weights (Figure 8) shows that the most diverse components were in the range of 1001 Da to 2000 Da and the second most diverse were in the range of 2001 Da to 3000 Da. This distribution suggests that this venom has a great diversity of short chain peptides (9–26 amino acid residues). In other members from the Vaejovidae family, an important number of HDP components were found containing short chain peptides composed by 13 to 22 amino acid residues. Their estimated molecular masses are found within the range of 1000 Da to 2000 Da [8,9]. From the 204 components identified in the fingerprint, only a few had a perfect match with the theoretical molecular weights predicted for each complete mature peptide (see Supplementary Table S1) of the translated transcripts. Four identical matches were found: SgeCatClc02 (MW 3774.41; RT 0–20), SgeHDPND202 (MW 4950.64; RT 220–240), SgeHDPND204 (MW 4792.4; RT 220–240), and SgeHDPND403 (MW 1625.94; RT 160–180).

#### 2.3.2. LC-MS/MS Analysis of the Digested Venom of *S. gertschi*

The soluble fraction of venom from *S. gertschi* was analyzed by LC-MS/MS. For MS raw data analysis, including dissociation by CID and HCD, we used the SEQUEST algorithm available in the software Proteome Discoverer and an in silico database including the predicted mature peptides of the 119 translated transcripts identified in the transcriptome. The LC-MS/MS analysis allowed confirmation of the presence of 24 tryptic peptide fragments, of which seven are ion channel toxins that corresponds to: two NaScTx (α-NaScTx and one β-NaScTx, also named SgeNaTAlp02 and SgeNaTBet02, respectively); two scorpines, which are members of the KScTx family (SgeKTxScr01 and SgeKTxScr02); and three toxins belonging to the CaScTx family, the liotoxins SgeCaTLio01, SgeCaTLio02, and the calcin SgeCaTClc02. Four peptides that correspond to the HDP family were also identified: NDBP2 (SgeHDPND204 and SgeHDPND202), NDBP3 (SgeHDPND301), and NDBP4 (SgeHDPND404). Additionally, five enzymes were identified, of which three of them belonged to the phospholipase family (SgeEnzPA204, SgeEnzPA206 and SgeEnzPA207), as well as one hyaluronidase (SgeEnzHya01) and one metalloprotease (SgeEnzMtP02). Finally, we identified two La1 peptides (SgeOthLa104 and SgeOthLa106) and one fragment with the annotation of a CAP superfamily protein (SgeOthCAP02) (see Table 2). Interestingly, five translated transcripts (c15440_g1_i1, c23802_g1_i1, c27313_g1_i1, c26154_g1_i1, and c26889_g1_i1) matched to MS data that do not have annotation in any database, neither as a transcript nor as a peptide. These fragments may correspond to novel components present in the venom that have not yet been reported.

### 2.4. Venom Enzymatic Activities

As described in the Material and Methods section, venom from the scorpion *T. serrulatus* and the snake *B. asper* were used as positive controls of enzymatic activities (hyaluronidase and phospholipase A2).

**Figure 9:**
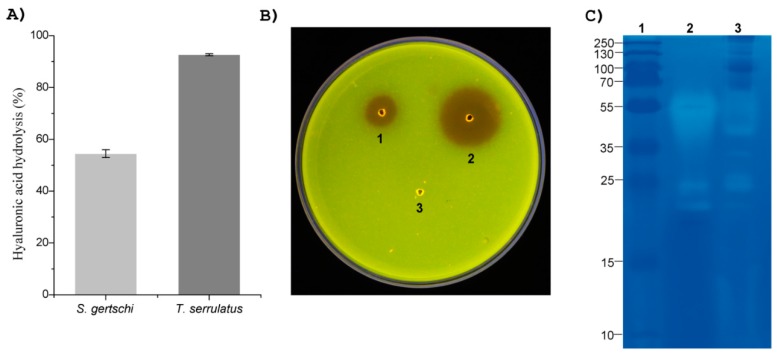
Enzymatic detected in the *S. gertschi* venom. (**A**) Hyaluronidase activity. The turbidimetric method at 595 nm was used for the determination of relative HA hydrolysis. Values represent the means ± SD from three independent experiments. (**B**) Phospholipase A2 activity, determined by the egg yolk agarose plate method. The wells contained the following samples: 1—*S. gertschi* venom, 2—*B. asper* venom (positive control), and 3—distilled water (negative control). (**C**) Proteolytic (gelatinolytic) activity revealed by a zymogram in a gelatin-containing PAGE. The indicated lanes in the gel correspond to: 1—Protein ladder (values in kDa), 2—*B. asper* venom (positive control), and 3—*S. gertschi* venom.

The hyaluronidase activity was measured using 40 µg of resuspended lyophilized venoms. The venom from *S. gertschi* hydrolyzed 54% of the HA substrate, while the positive control gave a 92% hydrolysis (Figure 9A).

The phospholipase A2 activity was evaluated using 20 µg of the resuspended lyophilized venom from *S. gertschi.* This amount of venom, after 12 h, caused a hydrolysis halo of 11 mm, as compared to 22 mm for the positive control (Figure 9B).

The presence of proteolytic enzymes in the venom was assessed by electrophoresis in a gelatin co-polymerized with the polyacrylamide gel. The venom from *S. gertschi* showed clear bands with relative molecular masses of c.a. 20 kDa, 25 kDa, 27 kDa, 35 kDa, 40 kDa, and 55 kDa, whereas in the venom from *B. asper* (positive control), protease-related bands were observed at 20 kDa, 25 kDa, and 55 kDa (Figure 9C). These clear bands demonstrated the hydrolysis of the substrate.

## 3. Conclusions

The transcriptome analysis of *S. gertschi* venom glands permitted the annotation of 119 sequences of putative proteins with diverse functions such as enzymes, ion channel toxins, HDP, protease inhibitors, and other venom components. Transcripts putatively coding for ion channel toxins were most diverse (19%), especially those coding for K^+^ channel toxins, which was a singularity of this transcriptome as compared to other previously reported vaejovid scorpion transcriptomes (that is: 13% in *T. atrox* [8] and 14% in *P. schwenkmeyeri* [9]). The LC-MS/MS analysis validated the adequacy of the transcriptome assembly and annotation. Major enzymatic activities predicted by the transcriptomic and proteomic analyses were experimentally demonstrated. That the venom LC-MS/MS analysis reported several proteins that correspond to unannotated transcript-derived peptides demonstrates that there are still some never-studied scorpion venom components of unknown activity, reinforcing the idea that the functional characterization of the scorpion venoms is far from exhaustive. Molecules with potentially relevant bioactivities are still waiting to be discovered. The sequence database generated in this work is certainly contributing to the knowledge on the general venom composition of the scorpions of the Vaejovidae family.

## 4. Materials and Methods

### 4.1. Biological Material

Scorpion specimens were collected in Ensenada Baja California, Mexico on August 2015 and August 2016. Permits for collection were issued by the Secretaría de Medio Ambiente, Recursos Naturales y Pesca (SEMARNAT) (Scientific Permits FAUT-0175 granted to Oscar Francke, see acknowledgements, and SGPA/DGVS/07805/16 given to Lourival Domingos Possani, Date of approval: 03 August 2016). Information available in the published literature allowed the identification of the scorpion under study [11]. The animals were maintained in plastic boxes with hideouts, with water *ad libitum* and fed with crickets. Five days prior to the RNA extraction procedure, the scorpions were milked by electrostimulation to deprive the glands from venom, and therefore stimulate the expression of the venom components, maximizing the RNA amounts. After milking, the scorpions were kept unfed until telson dissection. Eight specimens were euthanized to dissect the telsons, and the rest were deposited at the “*Colección Nacional de Arácnidos*” at the Biology Institute of the National Autonomous University of Mexico, in Mexico City.

### 4.2. RNA Extraction, RNA-Seq and Venom-Gland Transcriptome Assembly

Total RNA was isolated using the SV Total RNA Isolation System Kit (Promega, Madison, WI, USA), as in other studies (e.g., Santibáñez-López et al. [18,19]). The telsons from four male and four female specimens were dissected under RNAse-free conditions and pooled into a single tube containing the RNA lysis buffer (Promega, Madison, WI, USA). The samples were further processed as suggested by the kit manual, including the three-min 70 °C incubation step. The purified total RNA was recovered in nuclease-free water. A Nanodrop 1000 (Thermo Scientific, Waltham, MA, USA) was used for quantification of the purified RNA, and its integrity was confirmed using a 2100 Bioanalyzer (Agilent Technologies, Santa Clara, CA, USA).

Using 1 µg of the total RNA obtained, a complementary DNA (cDNA) was constructed, using the Illumina TruSeq Stranded mRNA Sample Preparation Kit (Illumina, Inc., San Diego, CA, USA), according to the protocol provided by the supplier. The Massive DNA Sequencing Facility in the Institute of Biotechnology (Cuernavaca, Mexico) was used for automated DNA sequencing. A Genome Analyzer IIx (Illumina, Inc., San Diego, CA, USA), using a 72 bp paired-end sequencing scheme over cDNA fragments ranging between 200–400 bp in size, was employed. Two fastq files (from forward and reverse reads) were generated. The quality of the raw reads was assessed with the FastQC program (http://www.bioinformatics.bbsrc.ac.uk/projects/fastqc/). No low-quality reads (below Q20) were obtained, so a quality-based trimming was not required. Only rRNA and adapter sequences were removed.

The reads were assembled *de novo* into contigs with the Trinity software (v. 2.0.3, GitHub, San Francisco, CA, USA), with the same parameters as reported before [9]. Basic statistics such as the number of ‘genes’, transcripts, and contigs were determined with the TrinityStats.pl script. Then, they were annotated with Trinotate (https://trinotate.github.io/ [93]). The pipeline and parameters used are detailed in a previous transcriptome report [9].

### 4.3. Transcript Nomenclature

Transcripts that were annotated as putatively coding for venom components were named in accordance with the nomenclature proposed by Romero-Gutiérrez et al. [8] and Cid-Uribe et al. [9]. The species’ identifier used was Sge, which stands for *S. gertschi*.

### 4.4. Bioinformatics

Multiple alignments of the sequences were found, and their similar sequences were obtained using the online version of Clustal Omega (https://www.ebi.ac.uk/Tools/msa/clustalo/). Alignments were manually edited with Bioedit (http://www.mbio.ncsu.edu/BioEdit/bioedit.html) and Adobe Photoshop CC 2017. The prediction of the signal peptide, propeptide, and mature peptide was performed using ArachnoServer (http://www.arachnoserver.org/spiderP.html), SignalP 4.1 Server (http://www.cbs.dtu.dk/services/SignalP/), and ProP 1.0 Server (http://www.cbs.dtu.dk/services/ProP/). For quantification of the GO terms, we used WEGO (http://wego.genomics.org.cn/). The translated protein sequences were analyzed for Pfam matches with the online server (https://pfam.xfam.org). The criteria that were used to annotate transcripts as putatively coding for scorpion venom components were those previously reported [8]. Figures were created using the ‘ggplot2’ package of the Rstudio suite (https://www.rstudio.com/).

### 4.5. Molecular Mass Determination of the Venom Components

The venom milked from the eight scorpion specimens was pooled, solubilized in water, and centrifuged at 15,000× *g* for 10 min. The protein concentration was estimated based on absorbance at λ = 280 nm, assuming that one unit is equal to 1 mg/mL of protein content. Five micrograms of soluble venom was automatically applied in a LC-MS/MS system composed of a nanoflow HPLC Dyonex 3000 and a mass spectrometry LTQ-Orbitrap-Velos, both from Thermo Scientific (San Jose, CA, USA). The fractionation of venom was carried out on an analytical capillary C18 column (100 mm, ID 75 µm), the mobile phases were 0.1% of formic acid (FA) in water as solvent A and 0.1% of FA in acetonitrile as solvent B, and the elution step was performed with a linear gradient of 5% to 80% solvent B during 290 min at a flow rate of 300 nL/min. The eluting ions peptides were detected using the positive ion mode, and the full scans were acquired in the Orbitrap mass analyzer from 200 *m*/*z* to 2500 *m*/*z* with 100,000 resolution and automatically deconvoluted each 20-min run by Extract raw files utility (Xcalibur software from Thermo Scientific, Waltham, MA, USA). For the analysis of the generated data, a deconvolution algorithm to transform a charge state series into a molecular mass was applied.

### 4.6. Identification of Proteome by Tryptic Digestion and LC-MS/MS Analysis

For the identification of proteins, 15 micrograms of soluble venom of *S. gertschi* was solubilized in 50 mM of NH_4_HCO_3_ reduced with 55 mM of dithiothreitol (DTT) for 30 min at 56 °C, and then alkylated with 10 mM of iodoacetamide (IAA). All of the reagents were acquired from Sigma-Aldrich (Saint Louis, MO, USA). The alkylated sample was digested for 18 h at 37 °C with trypsin from Promega (Madison, WI, USA). Eight micrograms of tryptic peptides were desalted using ZipTipC18 (Millipore, Billerica, MA, USA) and applied into the LC-MS/MS system. MS data acquisition was performed as earlier described by Cid-Uribe et al. [9]. MS data was searched using the Protein Discoverer 1.4 program (Thermo-Fisher Co., San Jose, CA, USA) against a database previously obtained from the venom gland transcriptomic analysis of the scorpion *S. gertschi*.

### 4.7. Venom Enzymatic Activities

The turbidimetric method was used to determine the hyaluronidase activity, in which a decrease in turbidity of a hyaluronic acid (HA) solution is correlated with this enzymatic activity [94]. The precise procedure followed was described previously [9]. Forty micrograms of the lyophilized soluble venoms of *S. gertschi* and *Tityus serrulatus* (positive control [95]) was used in the assays. The assays were performed in triplicate, and the results were expressed as a percentage of hydrolyzed HA with respect to the negative control (no venom added).

Phospholipase A2 activity was evaluated as described by Habermann and Hardt [96]. Twenty micrograms of the lyophilized soluble venoms from *S. gertschi* and the snake *Bothrops asper* (positive control [97]) was resuspended in 10 µL of distilled water and deposited into small wells in egg yolk agarose. Samples containing just water were used as negative control. Plates were incubated for 12 h at 37 °C. During incubation, the enzymes diffused into the agar, and the hydrolysis of the phospholipids resulted in a clear halo. The diameters of these areas were measured in millimeters.

Proteolytic activity was assayed using gelatin as the substrate in a polyacrylamide gel. Fifty micrograms of the lyophilized soluble venom of *S. gertschi* and 5 µg of the venom of the snake *B. asper* (positive control) was solubilized in 20 µL of non-reducing sample buffer and separated on a polyacrylamide gel cast with gelatin type A. The detailed procedure followed was reported previously [9]. The gelatinolytic (protease) activity is evidenced by clear zones in the gel after staining.

## Figures and Tables

**Table 1:** Molecular mass fingerprint of the soluble venom of *S. gertschi*. Monoisotopic molecular mass was considered for those components with MW inferior to 3000 Da, and an average molecular mass for those components with MW superior to 3 kDa.

Retention Time (Min)	Molecular Mass (Da)
0−20	444.26; 492.30; 673.37; 748.24; 775.41; 815.48; 888.44; 1018.52; 1105.66; 1158.64; 1234.61; 1245.64; 1374.74; 1434.69; 1460.76; 1484.79; 1573.83; 1633.82; 1694.86; 1731.90; 1832.95; 2290.88; 2350.31; 2452.07; 3464.44; 3565.49; 3616.63; 3648.56; 3701.77; 3773.73; 3928.89; 4052.58; 4361.81; 4544.96; 4744.10; 4815.13; 4879.28; 4882.28
20−40	2709.25; 3951.91; 4001.95; 4138.94; 4180.02; 4694.16
40−60	645.34; 683.30; 963.55; 1286.78; 1305.70; 1343.65; 1413.68; 1902.00; 2124.17; 3937.86; 4393.03; 5053.20; 5318.35
60−80	539.15; 722.39; 765.40; 836.46; 1004.54; 1193.63; 1264.68; 1408.59; 1643.90; 1750.81; 2036.03; 2103.22; 2229.20; 2411.28; 2467.34; 2580.42; 2629.31; 2757.39; 2855.56; 2837.54; 2984.55; 3338.73; 3350.48; 4068.67; 4348.99; 4444.81; 8029.68
80−100	707.35; 890.52; 978.51; 1518.92; 1647.01; 1744.06; 1859.09; 1972.17; 2561.42; 2950.62; 3274.43; 3421.02; 6230.62; 6418.87; 6568.88; 7088.10; 9023.88
100−120	1162.70; 1391.84; 1616.99; 1677.98; 1845.10; 1883.05; 2057.18; 2083.02; 2575.43; 3219.89; 3914.08; 6741.05; 6813.82; 7070.08
120−140	1380.75; 1495.78; 1582.81; 1683.86; 1721.82; 2248.22; 2361.30; 3001.49; 6457.78
140−160	804.39; 871.42; 1081.50; 1925.03; 2066.00; 3327.88; 3529.02; 6142.59; 6614.95; 6725.05
160−180	727.49; 876.43; 932.56; 1138.62; 1251.71; 1348.67; 1626.82; 1717.00; 1786.11; 1823.99; 2014.18; 2151.16; 2315.34; 2917.48; 3290.92; 6126.59; 6441.77; 6586.89
180−200	557.37; 989.52; 1046.54; 1119.63; 1567.83; 1603.92; 1807.94; 1888.12; 1956.00; 2146.18; 2485.44; 2674.46; 3031.66; 3310.88; 4256.28
200−220	599.40; 712.48; 1360.80; 1475.83; 1588.91; 1759.02; 2275.35; 2493.38; 2557.42; 2820.65; 3044.73; 3263.67; 3394.80; 3689.03; 4330.32; 4592.48; 4720.54; 4791.57; 4862.60; 4949.65; 12,869.20
220−240	657.36; 770.45; 883.51; 1027.60; 1096.62; 1185.67; 1298.75; 1438.81; 1525.84; 1838.02; 2371.36; 4428.40; 4652.41; 11,833.52; 12,386.79; 12,432.80
240−290	N/D

N/D: Not determined.

**Table 2:** The *de novo* sequences from the LC-MS/MS analysis that matched translated sequences from the transcriptome analysis. Lowercase letters are shown with amino acids with posttranslational modifications identified by the software.

Transcriptome ID	Score	Coverage	Protein Fragments	Xcorr of the Protein Fragments	Protein Type	Accession Number of the Reference Protein
SgeKTxScr02	1248.72	56.41	HGCLADFDVGGGCEQHCR	7.26	Potassium channel toxin. Scorpine-like peptide.	API81325.1
			KIQDAIDR	2.54		
			AWISEK	1.91		
SgeHDPND301	163.69	87.50	FWGFLGK	3.18	HDP. NDBP-3 family.	ALG64975.1
			TIPSLLGGSK	2.09		
SgeHDPND204	153.72	36.67	KAWNSNIGK	3.61	HDP. NDBP-2 family.	AGK88593.1
			AWNSNIGK	2.31		
SgeHDPND202	138.57	63.83	AWNSDIGK	3.59	HDP. NDBP-2 family.	ALG64975.1
			GIWGTIK	2.76		
			IGVTPSQAAS	2.70		
SgeEnzPA207	109.20	50.60	YGLSnTGSYTLLNcDcEK	6.04	Enzyme. Phospholipase A2.	API81335.1
			WIYFTAYSPK	2.85		
			cANPVGKWKADYK	1.38		
			EGWIKK	1.02		
SgeCaTLio02	65.14	42.86	DLPLSNEYETcVRPR	3.74	Calcium channel toxin. Liotoxin.	P0DJ08.1
SgeEnzPA204	64.99	36.4	TLLNcDcEEAFDHcLQTTADK	4.38	Enzyme. Phospholipase A2.	API81335.1
			TLLNcDcEEAFDHcLQTTADKLEGADKEDTK	3.99		
			IIQNYYFNIFK	3.18		
			cRmLnSTKEVAR	1.17		
SgeHDPND404	64.77	53.85	DLWNGVK	2.37	HDP. NDBP-4 family.	I0DEB3.1
SgeNaTBet02	49.29	47.54	GSSYGYcYGFGcYcEGLNDDVK	4.87	Sodium channel toxin. Beta.	P01491.3
			FcQSIcK	2.46		
SgeOthLa104	14.30	65.82	NVPGPVDAPFPDccPTSLcR	4.24	La1-like peptide	AOF40216.1
SgeEnzPA206	13.61	40.08	AFYFHLYGnGcYHVK	3.79	Enzyme. Phospholipase A2.	API81339.1
			cLDQVVDGTSWYDYHATLGLIK	1.31		
			SENGRGLR	1.09		
SgeOthLa106	13.36	33.33	TGQYLNEGEEWRDPNHcSIYQcR	3.82	La1-like peptide	API81334.1
SgeNaTAlp02	12.80	64.79	AmPPFGLPGGcFcPNIPK	3.14	Sodium channel toxin. Alpha.	Q5MJP5.1
SgeCaTLio01	12.17	88.57	NLPLSDEYGTcVRPR	2.96	Calcium channel toxin. Liotoxin.	P0DJ08.1
			cKPPLWcNPQQIcVYK	2.86		
SgeKTxScr01	10.51	71.08	GVDALTNLIPAPVIGGIVNK	4.70	Potassium channel toxin. Scorpine-like peptide.	P0C8W5.1
			VQELcAFNK	3.31		
SgeOthCAP02	8.09	48.96	STNDFIFYcDFSK	4.15	CAP superfamily	API81352.1
			VEHSGTAFWTIGmHMqFDQEMESTIKLEAYR	1.33		
			VLYTcNYGPAGNmQGGTmYIKGEPcSQcPK	1.21		
SgeEnzHya01	4.11	31.30	YFTDTTSVFDSK	2.43	Enzyme. Hyaluronidase.	API81375.1
			ILVNNK	1.68		
			ILVNNKESFVGDK	1.07		
SgeEnzMtP02	3.85	27.14	KFNYPHGTIK	2.15	Enzyme. Metalloproteinase.	AMO02513.1
			KEPKKPPVPK	1.19		
SgeCatClc02	2.27	24.24	ADcLPHLK	2.27	Calcium channel toxin. Calcin.	API81327.1
c15440_g1_i1	6.83	9.30	ELTLEDVGTILR	3.67	N/D	N/D
c23802_g1_i1	7.08	10.48	WYNDcIDYcDR	3.59	N/D	N/D
c27313_g1_i1	13.32	15.28	FGDITLLGPEVTNR	4.12	N/D	N/D
			IISSPFYK	1.21	N/D	N/D
c26154_g1_i1	7.74	10.53	LAELETIVALAK	3.85	N/D	N/D
c26889_g1_i1	94.33	20.29	EFFLGLLGLK	3.63	N/D	N/D
			IDPKEFFLGLLGLK	1.54	N/D	N/D

N/D: Not determined.

## Supplementary Materials

The following are available online at http://www.mdpi.com/2072-6651/10/9/359/s1, Table S1: Sequences of the 119 transcripts obtained from the transcriptome analysis from the *S. gertschi* venom gland that putatively code for venom-related peptides. Sequences were classified in accordance to Pfam and other previously reported sequences from venomous animals. ID reference protein: UniProt or NCBI accession numbers. In the amino acid sequences, the predicted signal peptides are underlined, the predicted mature peptides are indicated in bold typeface, and the predicted propeptides are shown in italics. The molecular weight only was calculated for the complete CDS. N/D: no determinate for partial CDS.
